# Necessity of tricuspid valve replacement in a case with iatrogenic rupture of anterior leaflet by pacemaker lead

**DOI:** 10.1186/1749-8090-8-S1-P23

**Published:** 2013-09-11

**Authors:** A Gurbuz, O Gokalp, U Yetkin, H Iner, I Yurekli

**Affiliations:** 1 Izmir Katip Celebi University Ataturk Training and Research Hospital, Department of Cardiovascular Surgery, Turkey

## Background

Although there are many complications after pacemaker implantation, iatrogenic perforation of the tricuspid valve followed by tricuspid regurgitation is extremely rare.

## Method

Our case was a 64-year-old male. He was implanted a VDD pacemaker (PM) at another institution 15 months before his admission to our clinic. Four months after that implantation, the pocket for generator was changed due to infection of the initial pocket. He was suffering from ulceration of the skin covering the generator and exposed leads. Our Department of Cardiology removed the generator and the leads.

## Results

Control transthoracic and transesophageal echocardiography revealed that proximal part of the silicon tube left from the ventricular lead was prolapsing from right ventricle into the right atrium. A mobile mass of 9x3 mm was also observed on this part. In addition, a severe tricuspid regurgitation was detected due to coaptation defect with a pulmonary arterial pressure of 55-60 mm Hg and dilated right cardiac chambers. Extraction of the silicon tube from right atrium using inflow occlusion on beating heart (IOBH) technique was planned. But, iatrogenic perforation of the anterior leaflet of the tricuspid valve was detected and cardiopulmonary bypass was established. Perforation of the anterior leaflet and near-total detachment of tricuspid valve were detected and therefore tricuspid valve was resected. Replacement with porcine bioprosthetic valve was performed. Postoperative period was event-free and our patient is recently under outpatient follow-up.

## Conclusion

Although lead extraction using IOBH technique is an exclusive therapy, conventional cardiopulmonary bypass should be established and valvular repair/replacement should be carried out in cases with coincidental diagnosis of major iatrogenic valvular complication as in our case.

